# A Third Supernumerary Tooth Occurring in the Same Region: A Case Report

**DOI:** 10.3390/dj11020049

**Published:** 2023-02-12

**Authors:** Tatsuya Akitomo, Yuria Asao, Yuko Iwamoto, Satoru Kusaka, Momoko Usuda, Mariko Kametani, Toshinori Ando, Shinnichi Sakamoto, Chieko Mitsuhata, Mikihito Kajiya, Katsuyuki Kozai, Ryota Nomura

**Affiliations:** 1 Department of Pediatric Dentistry, Graduate School of Biomedical & Health Sciences, Hiroshima University, Hiroshima 734-8553, Japan; 2 Center of Oral Clinical Examination, Hiroshima University Hospital, Hiroshima 734-8553, Japan

**Keywords:** dental anomalies, supernumerary tooth, surgical treatment, follow-up, childhood

## Abstract

The presence of a supernumerary tooth is one of the most common dental anomalies, and surgical treatment is often required to address this anomaly. Moreover, it may lead to malocclusion, and long-term follow-up is important to monitor its status. A 4-year-and-11-month-old boy was referred to our hospital for dental caries treatment. At 5 years and 5 months of age, a radiographic examination showed a supernumerary tooth (first supernumerary tooth) near the permanent maxillary left central incisor, and it was extracted 6 months later. Eighteen months after the extraction of the first supernumerary tooth, a new supernumerary tooth (second supernumerary tooth) was detected in the same region, which was extracted when the patient was aged seven years and seven months. Seven months later, another supernumerary tooth (third supernumerary tooth) was detected and extracted immediately. However, the permanent maxillary left central incisor did not erupt spontaneously even after 6 months. Therefore, surgical exposure was performed, and the central incisor erupted into the oral cavity. This report describes our experience with this patient with three metachronous supernumerary teeth and their management until the eruption of the permanent tooth. This report highlights the importance of long-term follow-up after supernumerary tooth extraction until the permanent teeth in that region have erupted completely.

## 1. Introduction

According to Das et al., (2012), the presence of supernumerary teeth in the long term may prevent the spontaneous eruption of adjacent permanent teeth [1]. In addition, Russell and Folwarczna (2003) reported that malocclusion, and eruption of the permanent teeth in abnormal locations may occur due to the presence of supernumerary teeth [2]. The occurrence of a supernumerary tooth is common [3], with one or two supernumerary teeth observed in most cases; Zhao et al., (2020) reported that three supernumerary teeth occurrences at the same site are extremely rare, even in a large study of tens of thousands of individuals [4].

We previously reported a case of the development of a new supernumerary tooth after the extraction of a previous supernumerary tooth in the same region [5]. However, after further follow-up, another supernumerary tooth was detected, and, eventually, three supernumerary teeth occurred in the same area. Recurrence of supernumerary teeth in the same region is very rare, and the occurrence of three supernumerary teeth in the same site at different times has not been reported previously. In this report, we describe the management protocol for the extraction of three supernumerary teeth and observation of a permanent tooth eruption.

## 2. Case Presentation

A 4-year-and-11-month-old Japanese boy with an intellectual disability was referred to the Pediatric Dentistry Clinic of Hiroshima University Hospital with the chief complaint of dental caries. He had been receiving dental checkups and treatment from a general practitioner since the age of 2. However, the dental caries was advancing rapidly, and he was referred to the hospital for caries management. With regard to the medical history, the patient was diagnosed with autism spectrum disorder and exhibited an intellectual delay of about a year. The medical and family histories were otherwise unremarkable, except for the presence of certain allergies.

**Figure 1:**
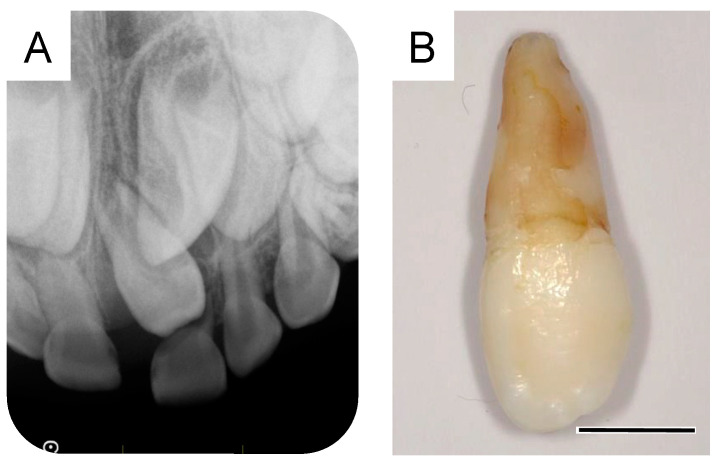
Extraction of the first supernumerary tooth. (**A**) Periapical radiograph of the first supernumerary tooth detected at the age of 5 years and 5 months. (**B**) Extracted first supernumerary tooth at the age of 5 years and 11 months. Scale bar: 5 mm.

At 5 years and 5 months of age, a supplemental supernumerary tooth (first supernumerary tooth) was detected near the permanent maxillary left central incisor (Figure 1A). This supernumerary tooth gradually erupted and was extracted 6 months later (Figure 1B). 

**Figure 2:**
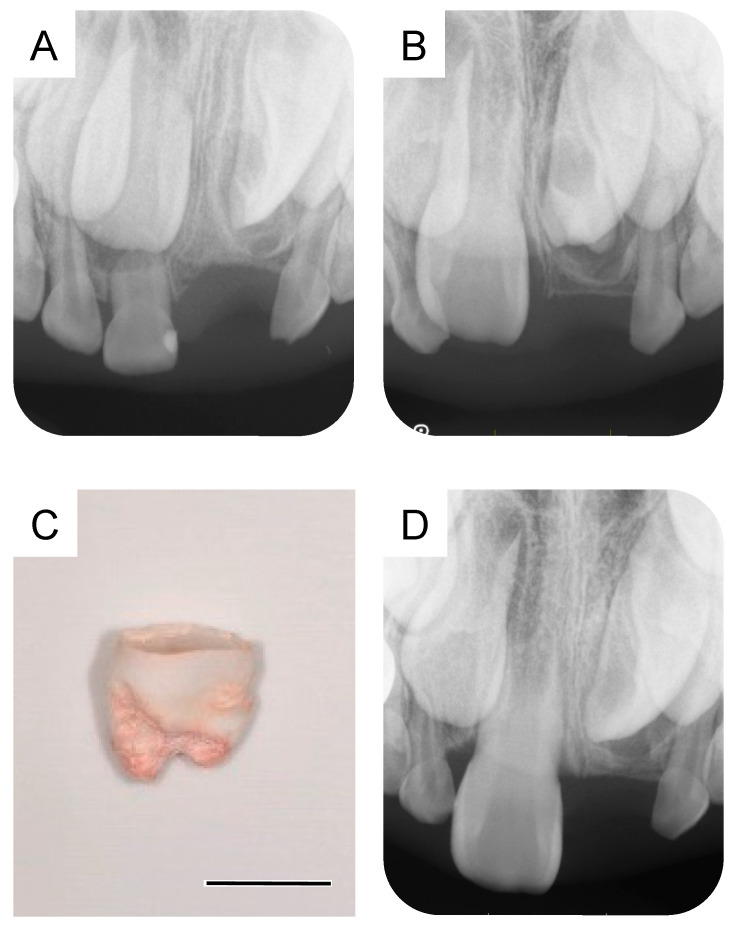
Extraction of the second supernumerary tooth. (**A**) Periapical radiograph after the extraction of the first supernumerary tooth at the age of 6 years and 3 months. (**B**) Periapical radiograph of the second supernumerary tooth detected at the age of 7 years and 5 months. (**C**) Extracted second supernumerary tooth at the age of 7 years and 7 months. Scale bar: 5 mm. (**D**) Periapical radiograph after the extraction of the second supernumerary tooth at the age of 7 years and 9 months.

A small radiolucency was observed near the crown of the maxillary left central incisor in the dental X-ray image taken during the first extraction. The dental X-ray images at 6 years and 3 months and 7 years and 5 months showed a progressive growth of this structure (Figure 2A,B), which was subsequently diagnosed as a supernumerary tooth (second supernumerary tooth). We extracted the second supernumerary tooth at 7 years and 7 months (Figure 2C). According to the general surgical procedure, debridement and suturing were performed. Radiographic examination at the age of 7 years and 9 months revealed an absence of any calcified tissue. However, a radiolucent image was observed around the germ of the permanent maxillary left central incisor (Figure 2D). Details of the first and second supernumerary teeth have been described in the previous report [5].

**Figure 3:**
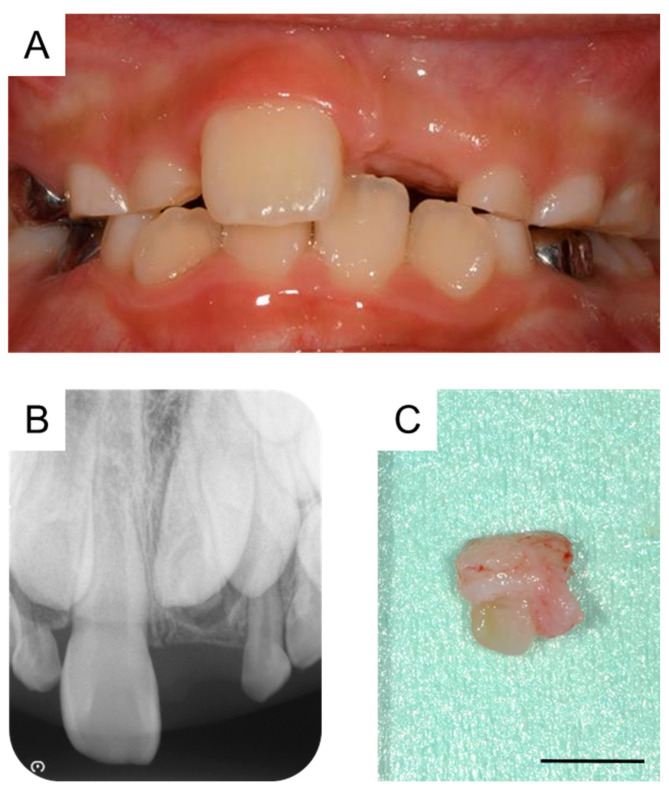
Extraction of the third supernumerary tooth. (**A**) Intraoral photograph at the age of 8 years and 2 months. (**B**) Periapical radiograph showing the third supernumerary tooth at the age of 8 years and 2 months. (**C**) Extracted calcified tissue, which was later diagnosed as the third supernumerary tooth. Scale bar: 5 mm.

**Figure 4:**
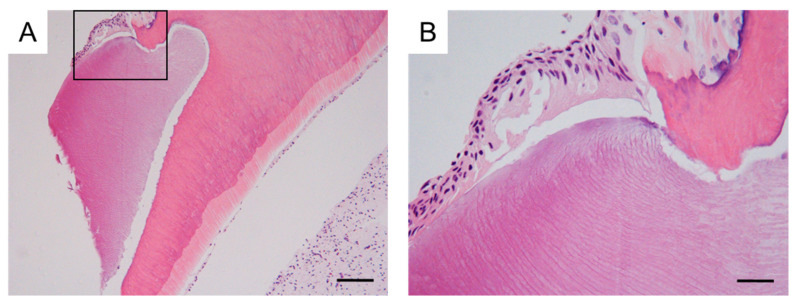
Histopathological evaluation of the third supernumerary tooth. (**A**) Representative histopathological examination images following hematoxylin and eosin staining of a tissue section of the third supernumerary tooth, including enamel, dentin, and pulp formation. Scale bar = 100 μm (×40). (**B**) High-magnification image of the box shown in (**A**). Enamel covered by reduced enamel epithelium and dentin is seen. Scale bar = 20 μm (×400).

By the time the patient was aged 8 years and 2 months, the permanent maxillary left central incisor had not erupted in the oral cavity (Figure 3A). A periapical radiograph revealed the presence of a calcified tissue near the crown of the permanent maxillary left central incisor (Figure 3B). Considering the difference in the eruption time of the bilateral permanent maxillary central incisors, we decided to take an immediate surgical approach. One month later, we surgically removed the calcified tissue and the bone covering the permanent maxillary left central incisor under physical restraint. The extracted calcified tissue measured 5 mm × 5 mm (Figure 3C). Histopathological examination showed the presence of a tooth-like structure with enamel, dentin, pulp, and reduced enamel epithelium, confirming the diagnosis of a third supernumerary tooth (Figure 4).

**Figure 5:**
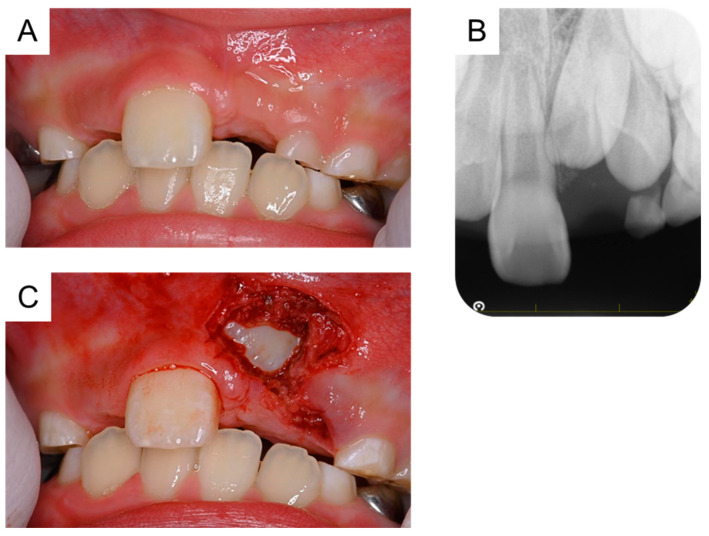
Fenestration of the maxillary left central incisor. (**A**) Intraoral photograph and (**B**) periapical radiograph after the extraction of the third supernumerary tooth at the age of 8 years and 9 months. (**C**) Intraoral image just after fenestration of the maxillary left central incisor at the age of 8 years and 9 months.

Orthodontic treatment was considered after the extraction of the third supernumerary tooth; however, the patient was uncooperative because of his intellectual disability. At 8 years and 9 months of age, a periapical radiograph revealed no change in the location of the permanent maxillary left central incisor (Figure 5A,B). Thus, under physical restraint and local anesthesia, the gingiva was surgically removed using an electrocautery device to expose the surface of the crown (Figure 5C).

**Figure 6:**
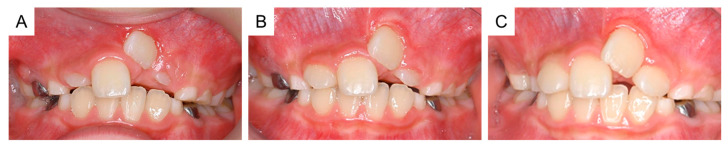
Intraoral photographs of the postoperative period. (**A**) One month after surgical exposure creation (8 years and 10 months of age). (**B**) Two months after surgical exposure creation (9 years and 0 months). (**C**) Four months after surgical exposure creation (9 years and 2 months).

**Figure 7:**
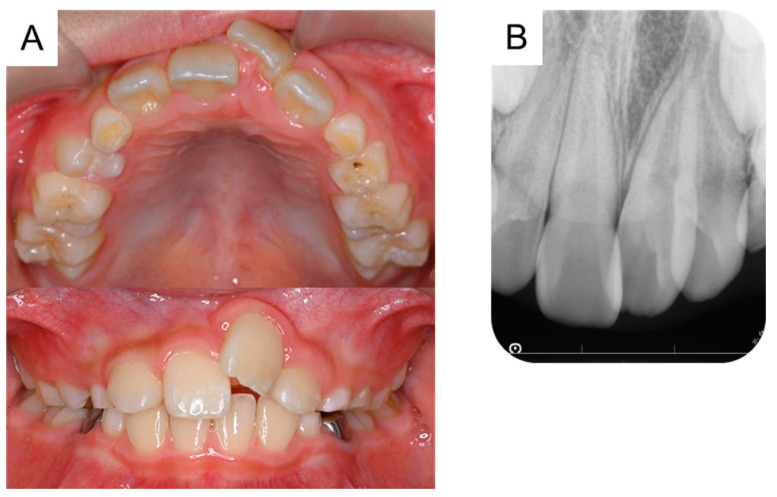
Intraoral photographs at regular checkups. (**A**) Intraoral photograph and (**B**) periapical radiograph obtained in the postoperative period at the age of 9 years and 7 months.

Following surgical exposure, the incisor gradually erupted in the oral cavity (Figure 6). At 9 years and 7 months of age, as confirmed by radiographic examination, no new supernumerary tooth had reoccurred. Intraoral examination revealed mesial inclination of the permanent maxillary left central incisor and a space shortage for the maxillary incisors (Figure 7). The patient is gradually showing an improvement in readiness for dental treatment. Therefore, long-term follow-up will be performed, and orthodontic treatment will be considered according to the wishes of the patient and parents.

## 3. Discussion

To the best of our knowledge, this is the first report of a case in which a third supernumerary tooth occurred after the extraction of the second supernumerary tooth. The treatment progression of the patient is shown in Table 1. In the present case, a small radiolucency was evident near the permanent maxillary left central incisor when the first supernumerary tooth was detected. However, it was impossible to diagnose it at that time. During follow-up examinations, calcification began and continued, and it was diagnosed as the second supernumerary tooth. The third supernumerary tooth was detected later; the three supernumerary teeth were eventually detected metachronously in the same region.

Yamashita et al., (1981) reported the formation of a root-like calcified tissue after the extraction of an immature permanent tooth [6], and they considered that this root was formed from the remaining Hertwig’s epithelial sheath derived from the extracted permanent tooth. Therefore, we hypothesized that the multiple supernumerary teeth-like structures in the present case could be independent supernumerary teeth or could be derived from the remnants of the roots of the extracted supernumerary tooth. In the present case, the first supernumerary tooth had a completely formed root at the time of extraction, and thus the second extracted structure could not have originated from the root of the first supernumerary tooth. Second, the second extracted tooth had not yet formed a root, and it is possible that the root-derived tissue was retained at the time of extraction. However, the third extracted structure was diagnosed as a new supernumerary tooth. Moreover, it was not derived from Hertwig’s epithelial sheath and included the enamel tissue, as confirmed on histopathologic evaluation. Therefore, all three structures extracted in our case were diagnosed as independent supernumerary teeth.

According to a report by Primosch et al., (1981), the treatment plan for embedded supernumerary teeth in the maxillary anterior region that cannot erupt is generally divided into an immediate and a delayed surgical approach [7]. Russell and Folwarczna (2003) and Primosch (1981) reported that an immediate surgical approach is often performed by around the age of 6 years and can improve tooth alignment and minimize the need for orthodontic treatment [2,7]. In contrast, Primosch (1981) and Shih et al., (2016) reported that a delayed surgical approach is performed around 8 to 10 years of age, when the roots of adjacent permanent anterior teeth are complete, to avoid the risk of harming the roots that are still forming [7,8]. Delayed surgical procedures have the advantage that the patient’s mental growth due to age can help facilitate treatment. In our present case, the first supernumerary tooth erupted and was extracted at the age of 5 years, wherein the X-ray revealed a small radiolucent image near the crown of the maxillary left central incisor (Figure 1A). An embedded structure may have been present before the initial surgery. However, it was difficult to diagnose the structure as a supernumerary tooth at this point. The subsequent dental X-ray images at 6 years and 7 years showed a progressive growth of this structure (Figure 2A,B), which allowed us to diagnose it as the second supernumerary tooth. Subsequently, at the age of 8 years, another developing structure was observed on the X-ray image and diagnosed as the third supernumerary tooth (Figure 3B). These embedded supernumerary teeth were extracted without further follow-up since their position likely inhibited the eruption of the maxillary left central incisor and they were in a low-risk position for damaging the roots of the adjacent permanent anterior teeth. The patient had autism spectrum disorder, was uncooperative while undergoing dental treatment, and was treated under restraint. In a case like ours, the timing of extracting the embedded supernumerary teeth needs to be carefully determined, taking into consideration the impact on the adjacent permanent teeth and the patient’s mental developmental status. This report also highlights the importance of follow-up after the supernumerary tooth extraction.

Russell and Folwarczna (2003) and Primosch (1981) reported that genetic factors have been implicated as one of the etiologies of supernumerary teeth [2,7]. According to a report by Lu et al., (2017), multiple supernumerary teeth are strongly associated with hereditary syndromic diseases, including cleidocranial dysplasia and Gardner’s syndrome [9]. In the present case, although the patient manifested an intellectual disability, he had no other abnormalities in his general condition and did not display a syndromic disease. Rai et al., (2020) and Palikaraki et al., (2019) reported that supernumerary teeth can be hereditary [10,11]; however, no family member in the present case exhibited any supernumerary teeth. Tominaga et al., (2021) reported that multiple supernumerary teeth may originate from the overgrowth of the tooth embryo or dental lamina [12]. Although the cause of the supernumerary teeth development in our case is unknown, these teeth may develop due to the overgrowth of the tooth embryo or dental lamina, and not due to heredity or familial origins.

The Japanese Society of Pediatric Dentistry has reported the eruption time of the permanent maxillary central incisors in Japanese males as 7.20 ± 0.67 years, which is considerably less than the age of the patient in this report [13]. Therefore, an eruption disturbance of the permanent maxillary left central incisor was suspected, and the third supernumerary tooth was extracted at the age of 8 years and 3 months. Because of the uncooperative nature of the patient, performing the orthodontic treatment using traction was difficult. Therefore, we decided to continue the follow-up. However, no tendency for spontaneous tooth eruption was observed for 6 months. The root of the permanent maxillary left central incisor was incomplete when the patient was aged 8 years and 9 months. We observed that the permanent incisor wobbled physiologically during the extraction of the third supernumerary tooth. Thus, we believed that the incisor had the ability to erupt spontaneously and decided to perform the surgical exposure. The permanent maxillary left central incisor erupted gradually after surgical exposure. 

An intraoral photograph at the age of 9 years and 7 months showed a mesial inclination of the permanent maxillary left central incisor and a space shortage for the maxillary incisors. Accordingly, orthodontic treatment such as a lateral expansion of the maxillary dental arch is required. We consider the treatment according to the wishes of the patient and parents. The goal of supernumerary tooth treatment is not only the extraction of the supernumerary tooth but also a proper eruption of the permanent tooth. Therefore, it is important to establish a long-term treatment plan, including orthodontic treatment.

This study had certain limitations. First, we previously reported the patient’s status until the extraction of his second supernumerary tooth [5]. Second, due to his intellectual disability, he was unable to cooperate with undergoing radiographic procedures, such as dental cone beam CT, and dental X-ray photographs could not be obtained. Third, we identified the permanent maxillary left central incisor intraorally after we removed the supernumerary teeth; however, we were unable to improve the alignment of the permanent maxillary left central incisor. We should treat the patient’s occlusion with attention to his cooperation level in the future.

## 4. Conclusions

We encountered a rare case of a third supernumerary tooth occurring in the same region after the extraction of the previous two supernumerary teeth. Multiple supernumerary teeth have been reported in a single patient; however, no report has documented the detection of three supernumerary teeth metachronously in the same region. In this case, the permanent maxillary left central incisor had not erupted spontaneously after the extraction of the third supernumerary tooth, and, eventually, surgical exposure was required. This report reconfirms that long-term follow-up is necessary and the need for a surgical approach, such as surgical exposure, should be considered if the permanent teeth are unable to erupt spontaneously after the extraction of the supernumerary tooth.

## Figures and Tables

**Table 1:** Treatment progression.

Age	Occurrences	Figure
4 years and 11 months	First visit to hospital	―
5 years and 5 months	First supernumerary tooth detection	Figure 1A
5 years and 11 months	First supernumerary tooth extraction	Figure 1B
7 years and 5 months	Second supernumerary tooth detection	Figure 2B
7 years and 7 months	Second supernumerary tooth extraction	Figure 2C
8 years and 2 months	Third supernumerary tooth detection	Figure 3A,B
8 years and 3 months	Third supernumerary tooth extraction	Figure 3C
8 years and 9 months	Surgical exposure	Figure 5A–C
9 years and 7 months	No recurrence of supernumerary tooth	Figure 7A,B

## Data Availability

Not applicable.
